# Optimizing Rehabilitation for Phantom Limb Pain Using Mirror Therapy and Transcranial Direct Current Stimulation: A Randomized, Double–Blind Clinical Trial Study Protocol

**DOI:** 10.2196/resprot.5645

**Published:** 2016-07-06

**Authors:** Camila Bonin Pinto, Faddi Ghassan Saleh Velez, Nadia Bolognini, David Crandell, Lotfi B Merabet, Felipe Fregni

**Affiliations:** ^1^Laboratory of Neuromodulation & Center for Clinical Research LearningPhysics and Rehabilitation DepartmentSpaulding Rehabilitation Hospital, Harvard Medical SchoolBoston, MAUnited States; ^2^Laboratory of Neuropsychology, IRCSS Istituto Auxologico Italiano,& Department of Psychology, NeuroMi - Milan Center for NeuroscienceUniversity of Milano-BicoccaMilanItaly; ^3^Spaulding Rehabilitation HospitalHarvard Medical SchoolBoston, MAUnited States; ^4^The Laboratory for Visual NeuroplasticityDepartment of Ophthalmology, Massachusetts Eye and Ear InfirmaryHarvard Medical SchoolBoston, MAUnited States

**Keywords:** cerebral cortex, clinical trial, electrical stimulation, electric stimulation therapy, factorial design, mirror therapy, non-invasive brain stimulation, transcranial electrical stimulation

## Abstract

**Background:**

Despite the multiple available pharmacological and behavioral therapies for the management of chronic phantom limb pain (PLP) in lower limb amputees, treatment for this condition is still a major challenge and the results are mixed. Given that PLP is associated with maladaptive brain plasticity, interventions that promote cortical reorganization such as non-invasive brain stimulation and behavioral methods including transcranial direct current stimulation (tDCS) and mirror therapy (MT), respectively, may prove to be beneficial to control pain in PLP. Due to its complementary effects, a combination of tDCS and MT may result in synergistic effects in PLP.

**Objective:**

The objective of this study is to evaluate the efficacy of tDCS and MT as a rehabilitative tool for the management of PLP in unilateral lower limb amputees.

**Methods:**

A prospective, randomized, placebo-controlled, double-blind, factorial, superiority clinical trial will be carried out. Participants will be eligible if they meet the following inclusion criteria: lower limb unilateral traumatic amputees that present PLP for at least 3 months after the amputated limb has completely healed. Participants (N=132) will be randomly allocated to the following groups: (1) active tDCS and active MT, (2) sham tDCS and active MT, (3) active tDCS and sham MT, and (4) sham tDCS and sham MT. tDCS will be applied with the anodal electrode placed over the primary motor cortex (M1) contralateral to the amputation side and the cathode over the contralateral supraorbital area. Stimulation will be applied at the same time of the MT protocol with the parameters 2 mA for 20 minutes. Pain outcome assessments will be performed at baseline, before and after each intervention session, at the end of MT, and in 2 follow-up visits. In order to assess cortical reorganization and correlate with clinical outcomes, participants will undergo functional magnetic resonance imaging (fMRI) and transcranial magnetic stimulation (TMS) before and after the intervention.

**Results:**

This clinical trial received institutional review board (IRB) approval in July of 2015 and enrollment started in December of 2015. To date 2 participants have been enrolled. The estimate enrollment rate is about 30 to 35 patients per year; thus we expect to complete enrollment in 4 years.

**Conclusions:**

This factorial design will provide relevant data to evaluate whether tDCS combined with MT is more effective than each therapy alone, as well as with no intervention (sham/sham) in patients with chronic PLP after unilateral lower limb amputation. In addition, this randomized clinical trial will help to investigate the neurophysiological mechanisms underlying the disease, which could potentially provide relevant findings for further management of this chronic condition and also help to optimize the use of this novel intervention.

**Trial Registration:**

Clinicaltrials.gov NCT02487966; https://clinicaltrials.gov/ct2/show/NCT02487966 (Archived by WebCite at http://www.webcitation.org/6i3GrKMyf)

## Introduction

Phantom limb pain (PLP) belongs to a group of neuropathic pain syndromes characterized by pain in the amputated limb [1-4]. In Western countries, the main reason for amputation is chronic vascular disease. In other parts of the world, civil wars and landmine explosions result in many cases of traumatic amputations in otherwise healthy people [5]. In the United States, 54% are due to vascular disease, 45% due to trauma, and less than 2% to cancer. According to the amputee coalition, there are approximately 2 million amputees in the United States and 185,000 amputations occur every year. From the individuals that had an amputation due to vascular disease, 50% will survive more than 5 years. Of the ones who had a lower extremity amputation due to diabetes, up to 55% will require the amputation of the second leg in 2 to 3 years.

PLP is experienced by 50% to 80% of the amputees. Although PLP may decrease or disappear over time, prospective studies indicate this is often not the case. Even 2 years after amputation, 59% of the patients reported PLP with only 5% to 10 % decrease in the intensity, exemplifying how it still remains a significant clinical problem that impairs quality of life [3,4].

### Mechanisms of Phantom Limb Pain

The precise mechanisms underlying development of pain in patients with limb amputation are not well elucidated. It has been demonstrated that long standing limb amputation can cause structural reorganization of the brainstem, thalamic nuclei, or the somatosensory cortex leading to maladaptive plastic changes [6-11]. Given the high concordance between motor and somatosensory plasticity, it is reasonable to assume that reorganization of the somatosensory cortex can also be detected in the motor cortex [12].

After an upper limb amputation, either shrinkage of the upper-limb region or expansion of the surrounding areas (lip/facial) is found in the primary somatosensory (S1) and motor (M1) cortex [6,8,10,13] *.* Using functional magnetic resonance imaging (fMRI), Lotze et al showed that the shift in the lip representation into the primary motor and somatosensory cortex is correlated with the amount of PLP [14,15]. Cortical reorganization secondary to an amputation additionally involves a decrease of GABA activity and an increased excitability of the corticospinal neurons over M1 [16-18] *.* These findings led to the current view that this reorganizational change represents a main pathophysiological mechanism of PLP [8,15,19,20] *.* Current rehabilitative therapies to treat PLP do not take into account such maladaptative plastic changes. An ideal therapeutic approach to treat PLP should aim to modulate and reverse the maladaptive plastic changes involved in the development of chronic PLP [21].

### Transcranial Direct Current Stimulation and Mirror Therapy

In this context, given that current options for pain treatment have insignificant or no effect on brain plasticity, the investigation of alternative approaches such as neuromodulation techniques can be used not only to alleviate pain but also to revert maladaptive plasticity. One candidate to promote plastic changes is transcranial direct current stimulation (tDCS). tDCS delivers a low intensity current that can modulate (facilitate or inhibit) spontaneous neuronal activity, its long term effects are likely to be mediated by mechanisms of synaptic long term potentiation and depression affecting neuroplasticity [22,23].

Recent studies have confirmed the therapeutic potential of tDCS in treating PLP. In 2013, Bolognini et al showed that a single session of anodal tDCS (2 mA, 15 min) targeting M1 induced a selective short-lasting decrease of PLP [24]. In addition, the same group showed the pain relief cumulative effects of tDCS with repeated sessions. After 5 consecutive days of anodal tDCS over M1 (1.5 mA, 15 min), participants experienced sustained decrease in PLP which lasted for 1 week after the end of the treatment, along with enhanced control of phantom limb movements [24,25]. These studies point out the preliminary yet promising role of tDCS in relieving PLP. The next step in this investigation would be to combine tDCS with a behavioral intervention. The learning of new skills (that is accompanied by behavioral changes) is linked to changes in neuronal activity and excitability [26]. They might reflect changes in synaptic strength, for example, N-methyl-D-aspartate (NMDA) receptor-dependent long term potentiation (LTP) [27].

Soler et al [28] conducted a factorial trial testing the combined effects of tDCS and visual illusion to treat patients with chronic neuropathic pain associated with spinal cord injury. The combination of tDCS and visual illusion was associated with the greatest pain reduction as compared to the either therapy alone. The results demonstrate and provide important preliminary data to support the rationale of this trial.

Therefore, combining tDCS with a behavioral intervention may optimize PLP rehabilitation. Mirror therapy (MT) seems to be the optimal behavioral intervention to activate sensorimotor cortex as shown by several studies [14,29-31]. Ramachandran et al (1996) were the first to describe the use of MT in order to evaluate its effects on phantom limb sensation in 10 upper limb amputees [32]. Foell et al [14] found a 27% decrease on a visual analogue scale (VAS) in 13 patients with unilateral upper limb amputation and chronic PLP after 4 weeks (15 min daily) of MT training (size effect=0.52). In addition, they found a relationship between the pain change after MT and a reversal of dysfunctional cortical reorganization in S1. In a pilot study involving 40 patients with PLP and unilateral amputation, Darnall et al [30] showed a significant reduction in average pain intensity at 1 and 2 months after home MT (25 min daily). There are also promising results from case reports and randomized clinical trials on the effectiveness of MT as a pain intervention in patients with PLP following amputation of upper or lower limbs [29,30]. However, the response to MT is usually heterogeneous, with treatment’s gains variable across individuals. Considering this heterogeneity and the fact that the analgesic effects of MT are not yet elucidated, it would be reasonable to combine it with a top-down cortical intervention, such as tDCS, aiming to improve its analgesic effect. Therefore, combining these two interventions could optimize the effects of each therapy alone, resulting in cortical changes and an efficacious and long lasting relief from PLP.

In summary, there is a great unmet need for non-invasive treatments for chronic PLP. In this protocol, we will test a novel rehabilitation approach combining a behavioral therapy (MT) with a method of brain modulation (tDCS) to treat and investigate the mechanisms of PLP.

### Aims and Hypotheses

#### Primary Aim

The primary aim of this clinical trial is to perform a comparative analysis of the efficacy of tDCS and MT as a rehabilitative tool for the management of chronic PLP in unilateral lower limb amputees.

#### Secondary Aim

The secondary aim of the study is to examine the mechanisms underlying PLP using two neurophysiological techniques. Single-pulse and paired-pulse transcranial magnetic stimulation (TMS) will be utilized to assess cortical mapping and cortical excitability changes associated with cortical reorganization. In addition, fMRI will be employed to assess brain changes, including the quantification of maladaptive cortical reorganization.

#### Hypotheses

We hypothesize that the combination of tDCS and MT will achieve greater effects when compared with the isolated use of either tDCS or MT, as well as with the sham tDCS combined with sham MT with regard to improvement (greater pain reduction) of chronic PLP, as indexed by the VAS scale in participants with unilateral lower limb amputation.

Our second hypothesis is that the combined group (tDCS and MT) will have a greater activation than any therapy alone and the no therapy group (sham tDCS and covered mirror) in the TMS and fMRI evaluations. In addition, neurophysiological and hemodynamic changes will be correlated with pain reduction.

## Methods

### Study Design

**Figure 1:**
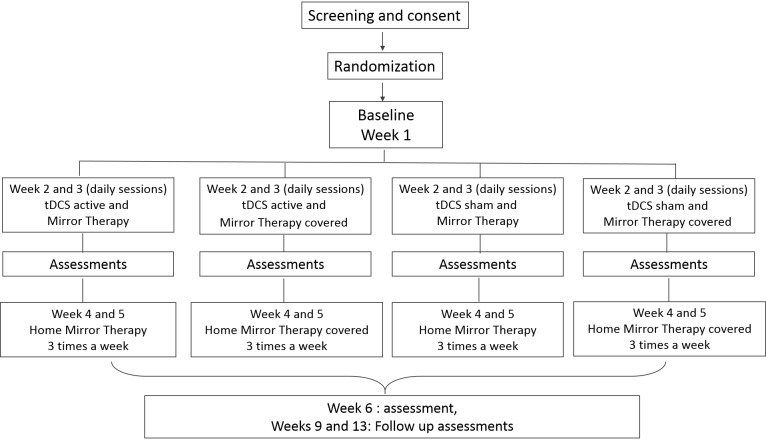
Flow chart of the study based on CONSORT criteria.

A prospective, randomized (allocation ratio 1:1:1:1), placebo-controlled, double-blind, factorial superiority study will be carried out (Figure 1).

### Study Setting

Patients will be recruited from the Limb Loss Clinic of the Spaulding Rehabilitation Hospital/Network and additional recruitment around the Boston, MA area. All study procedures will be performed at the Spaulding Neuromodulation Center in the Spaulding Rehabilitation Hospital, Charlestown, MA, USA.

### Eligibility Criteria

The eligibility criteria (inclusion and exclusion) for the study are shown in Textbox 1. Since the safety of tDCS in the pregnant population (and children) has not been assessed, pregnant women (and children) will be excluded. Women of child-bearing potential will be required to take a urine pregnancy test during the screening process.

**Inclusion and exclusion criteria.**

Inclusion criteria

- Able to provide informed consent to participate in the study
- Subject is older than 18 years
- Unilateral lower limb amputation
- 3 months of PLP after the amputated limb has completely healed
- Average pain of at least 4 on a numeric rating scale (NRS), ranging from 0 to 10 in the previous week
- If the subject is taking any medications, dosages must be stable for at least 2 weeks prior to the enrollment of the study

Exclusion criteria

- Pregnancy or trying to become pregnant in the next 2 months
- History of alcohol or drug abuse within the past 6 months, as self-reported
- Presence of the following contraindication to tDCS and TMS
  - Ferromagnetic metal in the head (eg, plates or pins, bullets, shrapnel)
  - Implanted neck or head electronic medical devices (eg, cochlear implants, vagal nerve stimulator)
- History of chronic pain previous to the amputation
- Head injury with post-traumatic amnesia for greater than 24 hours, as self-reported
- Unstable medical conditions (eg. uncontrolled diabetes, uncompensated cardiac issues, heart failure or chronic obstructive pulmonary disease)
- Uncontrolled epilepsy or prior seizures within the last 1 year
- Suffering from severe depression (as defined by a score of >30 in the Beck Depression Inventory)
- History of unexplained fainting spells or loss of consciousness as self-reported during the last 2 years
- History of neurosurgery, as self-reported
- MT within 3 months prior to enrollment

### Interventions

#### Transcranial Direct Current Stimulation

tDCS will be performed during the MT session, as this technique may facilitate behavioral changes by enhancing neuroplasticity and increasing functional connectivity. The Soterix Medical 1×1 tDCS stimulators device (Soterix Medical Inc.) will be utilized. This device sends a low-level current from the positive electrode (anode) to the negative electrode (cathode). During tDCS, low amplitude direct currents will be applied via scalp electrodes and penetrate the skull to enter the brain. Direct current will be transferred by a saline soaked pair of surface sponge electrodes (35 cm^2^) and delivered by a specially developed, battery-driven, constant current stimulator with a maximum output of 10 mA.

The tDCS device can be used with codes that correspond to active or sham stimulation, allowing a truly double-blind procedure. Participants will receive daily stimulation sessions with active or sham anodal tDCS for 10 days (5 days each week). Participants will be allowed to reschedule up to 3 stimulation visits (maximum of 2 consecutives). During each active anodal tDCS session, an anodal electrode will be placed over M1, contralateral to the amputation side and the cathode over the contralateral supraorbital area and tDCS will be applied for 20 minutes at 2 mA [24]. For the sham tDCS, the same montage of electrodes used for the active stimulation will be applied; however, current will be applied only for the first 30 seconds of the 20 minutes session. This is a reliable method of sham stimulation as sensations arising from tDCS treatment occur only at the beginning of application [33].

#### Mirror Therapy

For the active MT sessions, participants will be asked to perform movements (15 minutes daily) using the unaffected limb while watching its mirrored reflection superimposed over the affected limb. During MT, participants will be asked to consciously relate the movement observed in the mirror to their phantom limb and to keep their attention focused on the task. Instructions will be explained verbally, demonstrated by a therapist, and performed by the subject in front of the therapist during the first 2 weeks (the MT sessions will be scheduled at the same time as the tDCS sessions). After the training, participants will continue MT everyday for 2 more weeks at home. Participants will be instructed to stop MT if it intensifies their pain, and to document if this happens. For the sham MT (covered MT), participants will be asked to perform movements in the same way as the active group but with a covered mirror.

### Outcomes

#### Evaluation and Follow-Up

The participants in each group will be evaluated by an experienced researcher in the evaluation procedures and blinded to which group (active vs sham tDCS) each participant belongs. The following 14 evaluations will be carried out: (1) evaluation 1 will be carried out one week prior to the intervention, (2) evaluations 2-11 will occur before and after the intervention, (3) evaluation 12 will take place right after the home-based MT is finished, (4) evaluation 13 will take place 4 weeks after the home-based MT is finished, and (5) evaluation 14 will take place 8 weeks after the home-based MT is finished.

#### Pain Assessment

Pain assessment will be indexed by the VAS for pain. This scale is commonly used to obtain self-reported ratings of pain level on a visual scale (ie, unbearable to none). Participants will rate the intensity of their PLP from 0 (indicating no pain at all) to 10 (indicating the worst pain felt). They will also report the frequency of PLP paroxysms, when PLP clearly increases above the background level from 0 (never during the day) to 10 (very frequently) [24,25,34]. This colored VAS will be used, from green (at 0) to red (at 10), as a visual indicator of pain. This assessment tool is frequently used in research studies evaluating pain levels [24,25,29,34-37]. VAS will be used to measure stump pain, non-painful phantom limb sensation, phantom movements, and phantom limb telescoping [25,34]. In addition, an adapted version of the Groningen Questionnaire after Arm Amputation will be administered. This questionnaire was originally meant to obtain information concerning complaints that may be developed after arm amputation and an adaptation of the current arm version was developed to assess participants with lower limb amputation. This questionnaire has been used in several clinical trials assessing PLP [38].

A pain and medication diary will be filled out daily by each participant during the total duration of the trial. This assessment tool will help to monitor daily changes in pain levels, medication dosage information, as well as safety. Participants will be asked to record the number of PLP paroxysms (ie, when PLP clearly increases above the background level) on a daily basis using a pain diary. In addition, the participants will record the intensity of the strongest episode as well as non-painful phantom limb sensation, phantom movements and stump pain on different colored VAS included in the diary. Moreover, participants will record their current medications and dosages daily in a pain medication diary, until completion of the study.

#### Neurocognitive and Psychological Assessments

Participants will undergo assessments of neurocognitive and psychological aspects such as depression or anxiety. In the case of depression the subjects will be assessed with the Beck depression inventory [39]. This self-reported inventory consists of 21 multiple choice questions and is a widely used method to classify depression severity. It assesses for the presence of several symptoms related to depression, such as irritability, hopelessness, and decreased cognitive performance. Physical symptoms such as weight loss and fatigue are also included. This instrument has been used previously to evaluate depression severity in patients with PLP [40], as well as in other chronic pain conditions [28,41,42]. With respect to anxiety, participants will be assessed with the Beck anxiety inventory [39]. This self-reported inventory consists of 21 multiple choice questions about the participant's overall “feelings” during the previous week. It is designed for an age range of 17 to 80 years old. Each question has the same set of 4 possible answer choices, arranged in columns and answered by marking the appropriate one with a cross [39].

In order to assess potential cognitive decline, participants will undergo evaluation with the Mini Mental State examination (MMSE). This is a sensitive, valid, and reliable 30-point questionnaire that is used extensively in clinical and research settings to measure cognitive abilities. It will be used as a baseline evaluation [43].

#### Quality of Life, Safety and Adverse Effects Assessments

Each participant will undergo assessments to evaluate changes in quality of life and safety before and after the intervention. Quality of life will be assessed using the Short Form Health Survey (*SF-36*) [44]. SF-36 is used as a measurement of quality of life and provides a profile of functional health and well-being scores. It is also used as a psychometrical index of physical and mental health. This instrument is widely used as a quality of life assessment in patients after an amputation and those suffering from PLP [44-46]. In addition, the Stroop test will be performed. In this test, participants are presented with names of colors written in the same color or in a different color, thus on the one hand the word names a color (red) and is written in another color (blue). In this task, the automatized behavior (reading) is in conflict with the desired response (naming the color). The subject has to inhibit and/or suppress the automatic response of reading and naming the color the word is written in. The Stroop is one of the most commonly used tools for determining attentional problems and to assess executive function and working memory [47,48]. Here, the Stroop test will be used to assess cognitive changes from baseline to post-treatment and follow-up visits. Furthermore, the Patient’s Global Impression of Change scale will be applied in order to evaluate the participant's perception of change (if any) in the activity limitations, symptoms, emotions, and overall quality of life after their participation in the intervention visits of the trial [49].

#### Neuroimaging Study and Analysis

fMRI will be used to quantify patterns of activation associated with maladaptive cortical reorganization before and after treatment of each participant. Structural and functional imaging data will be acquired on a 3 Tesla Philips Achieva System (Best, the Netherlands) with a 32-channel phased array coil. Structural T1-weighted scans will be acquired using a turbo spin echo sequence (TE = 3.1 ms, TR = 6.8 ms, flip angle = 9°, voxel size 0.98 x 0.98 x 1.20 mm, no slice gap, acquisition matrix 256 x 254). Functional scans will be acquired with a single-shot EPI sequence (TE 28 ms, TR 2000 ms, flip angle 90 deg, and 3 mm isotropic resolution with no slice gap). Two functional runs will be collected each lasting 360 seconds in duration (see below for details regarding task design and contrasts of interest).

A repeated measures design (baseline and post-treatment) will be conducted for each participant. Additional baseline data will be obtained for secondary correlations. In addition, a sensitivity analysis testing the comparison of post-treatment will be conducted.

This design is based on previous work investigating the task-based activation of cortical motor networks associated with the observation and imagination of lower limb movements [50]. The task conditions will consist of the participant actively moving their non amputated limb (flexion and extension) at a predetermined frequency, followed by the same leg movement, but now the participant will be able to observe the image of his/her leg moving in a mirror (presented through an online video). These two conditions will be interleaved by a rest period of equal length in which the participant will be instructed to remain immobile. The following 3 conditions will therefore be investigated: (1) movement of the leg (MOV-LEG), (2) movement of the leg observing the mirror (MIR-LEG), and (3) rest condition. For the MOV-LEG, the participants will perform movements of the non amputated leg and each participant will be instructed about the type and pace of the movements. For the MIR-LEG, the participants will perform the same movements from the previous condition, looking at the mirror image of the intact leg in an online video. Again, each participant will be instructed about the type, pace, and video. During the rest period, the participants will rest and will be instructed to not perform any kind of movements. Each of the 3 conditions will have the same length (20 seconds). The fMRI session will have 4 runs each containing 6 blocks (6 repetitions of each condition).

For the analysis, the region of interest (ROI) will be selected. The ratio of activation between the MOV-LEG and MIR-LEG (in the respective ROI – contralateral to the respective leg) will be determined previously. Using this coefficient, a comparison across the 4 different conditions will be performed. For this sub study, a standard safety screening questionnaire will be administered prior to participation by the attending technician or study investigator (NINDS CDE) [51,52].

#### Transcranial Magnetic Stimulation

Transcranial magnetic stimulation (TMS) will be used to assess cortical excitability and cortical reorganization. For the TMS assessments, we will use Magstim Bistim2 stimulator and a figure 8 coil (Magstim Company LTDA, UK). The most distal muscle will be studied, in the case of the presence of the quadriceps we will place the electrodes over the rectus femoris, another pair of electrodes will be placed in the first dorsal interossei muscle (FDI), and a ground electrode will always be placed over the participant’s distal prominence of the ulna bone. Electromyogram (EMG) recordings will be processed using Powerlab 4/30 (ADinstruments, Colorado Springs, CO, USA) with a band pass filter of 20-2000 kHz. Offline analyses will be performed using LabChart (ADinstruments, Colorado Springs, CO, USA). First, head measures will be taken to identify the approximate spot of the motor cortex (using the vertex as the reference) [53]. Then, the TMS coil will be held over the motor cortex at an angle of 90 degrees with respect to the sagittal line of the head. The hotspot will be determined by carefully eliciting the most stable and highest *motor-evoked potentia* l (MEP) amplitudes over the rectus femoris [54-56]. The best location will be marked with a pen on a swim cap, which will be worn by each of the participants. Resting motor threshold (rMTh) will be determined by eliciting 3 of 5 motor-evoked potentials (MEPs) with minimal peak-to-peak amplitude of 100 μV (according to Rossini et al) [57]. Changes in cortical excitability will be assessed by evaluating the MEP; to assess this aim 10 MEPs will be recovered for each hemisphere using 120% of the rMTh [53].

TMS measurements will include short-interval intracortical inhibition (SICI) and intracortical facilitation (ICF) using the paired-pulse technique [22]. For paired-pulse measurements, the first stimulus will be set to 80% of the individual rMTh, and the second stimulus to the individual 120% of the MEP intensity at inter-stimulus interval of 2, 3, 6, 9, 10, and 12 ms. Ten recordings of each inter stimulus interval protocol will be randomly elicited (total of 60 measures). Offline analyses will include measures of peak-to-peak amplitude, the area-under-the-curve of all MEPs, and the relative duration of cortical silent periods (CSPs) (time from last MEP until normal muscle activity was re-achieved). For the cortical mapping measures, 8 stimulations at 120 % of rMTh intensity (posterior to anterior current) will be delivered to each of 15 sites forming a 3x5 grid, with a constant 1.5 cm distance between sites, over M1 [22]. At each stimulation site, the peak-to-peak amplitudes of the recorded MEPs will be measured and averaged offline. The map center of gravity (CoG) will be computed for the medio-lateral (x) coordinates using the formula:

CoGx = (Σxi *MEPi) / ΣMEPi (1)

where MEPi represents the mean amplitude of the MEPs produced at one site. The sum of the average MEP amplitude will be calculated for each active site, where an active site was defined as a site at which the mean MEP amplitude was at least 0.05 mV.

### Participant Timeline

**Figure 2:**
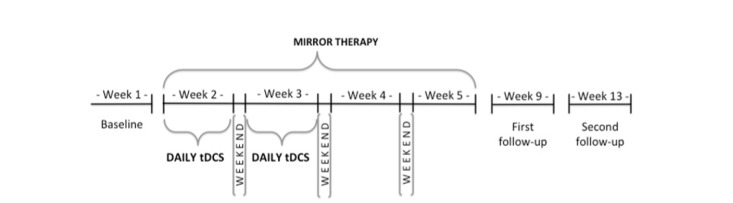
Schematic view of the experiment time points.

The study will take place for 13 weeks (Figure 2). All study procedures will be performed at the Spaulding Rehabilitation Hospital (Neuromodulation Center). Each participant will undergo 15 visits, described in Textbox 2.

**Study timeline.**

Visits

- Visit 1: Consent and screening
- Visit 2: Baseline
- Visits 3-12: Intervention visit
  - After visit 12, the participants will continue MT (alone, without tDCS) at home for 2 more weeks
- Visit 13: End of home intervention (MT)
- Visit 14: Follow-up 1, 4 weeks after visit 13
- Visit 15: Follow-up 2, 8 weeks after visit 14

The participants randomized to receive sham tDCS will have the opportunity to enroll into an open label portion of the study at the conclusion of their participation in the randomized portion of the trial.

### Study Sample

We will recruit 132 subjects with PLP of traumatic etiology; congenital or diabetic amputees will be excluded since these patients may have different neuroplastic profiles. Participants will need to meet all of the inclusion criteria and none of the exclusion criteria.

### Sample Size Calculation

The sample size was calculated utilizing STATA 11 program. We based our calculation on a study carried out by Soler et al (2010) entitled “Effectiveness of transcranial direct current stimulation and visual illusion on neuropathic pain in spinal cord injury” [28]. Using VAS as continuous outcome for the calculation and considering results from this study, a mean (SD) of 5.2 (1.5) in the experimental group (tDCS and visual illusion) and 6.4 (1.6) in the control group (visual illusion), and using a bidirectional alpha of .05 and power of 80%, we would need a total sample of 108 participants (27 in each group). In addition, estimating a conservative attrition rate of 20%, our sample size would be 132 participants. Although we calculated the sample for our primary aim, it is important to underscore that the power calculation will also be adequate for our secondary aim measuring neurophysiological outcomes. In fact, neurophysiological outcomes usually have less variability and thus needs smaller sample sizes to show significant differences. As shown in our preliminary data and data from other studies [55,58], effect sizes from TMS data will be larger and for the fMRI, we expect blood oxygen level dependent (BOLD) changes in sensory motor cortex around 3% to 5% (as shown by previous studies [50]), therefore a sample of 7 to 8 participants will be enough to detect significant changes.

### Recruitment

Individuals with chronic PLP after a unilateral lower limb amputation will be primarily recruited through the Limb Loss Clinic at Spaulding Rehabilitation Hospital/Network in Charlestown, MA. The Amputee Program at Spaulding Rehabilitation Hospital has more than 120 in-patients with amputations per year, and its Limb Loss Clinic has more than 200 amputees with PLP. Spaulding Rehabilitation Hospital was the primary rehabilitation center that received patients with traumatic amputations from the Boston Marathon Bombings. In addition, we will approach colleagues at the other Harvard teaching hospitals, including Brigham and Women’s Hospital (BWH) and Massachusetts General Hospital (MGH), and outpatient clinics in the greater Boston area.

### Randomization

Once eligibility and consent have been approved and completed, randomization will occur using the randomized list generated by an automatic Web-based randomization program. Participants will be randomly assigned to 1 of the following 4 groups: (1) Group 1 will receive active tDCS and MT, (2) Group 2 will receive sham tDCS and MT, (3) Group 3 will receive active tDCS and covered MT, and (4) Group 4 will receive both sham tDCS and covered MT.

The participants randomized to receive sham tDCS will have the opportunity to enroll into an open label portion of the study at the conclusion of their participation in the randomized portion of the trial.

Participants will be randomly assigned to 1 of the 4 groups in a 1:1:1:1 allocation ratio. We will use stratified randomization methods with random block sizes of blocks of 4 and 8. Stratification will be based on the participant’s baseline pain levels using 2 strata: less than or equal to an average 6 in VAS or greater than 6 in VAS). The randomization order will be kept in sealed envelopes; therefore participants will get their assignment according to the order of entrance in the study (for instance, participant 1 will be assigned the first envelope that will contain his/her assignment according to this block randomization list).This process will be carried out by a member of the research team who is not involved in the recruitment process or development of the study.

### Sequence Generation

Eligible participants will be randomized based on an allocation sequence generated by an independent person not involved in the study through a true randomization process.

### Allocation Concealment

A series of numbered, sealed, opaque envelopes will be used to ensure concealed allocation. The order of entrance to the study will determine the allocation of the participant.

### Implementation

A researcher not involved in the study will be in charge of the allocation.

### Blinding

Participants will be blinded by receiving sham tDCS stimulation with the same electrode montage as the active group. All trial researchers involved in the analysis and collection of data will be blinded to the treatment allocation group until after analyses are performed at the completion of the trial.

### Blinding Assessment

The tDCS blinding questionnaire will be performed after the stimulation session. Each participant will complete a questionnaire to determine if the blinding methods were effective [59].

### Data Collection Methods and Management

Data forms and questionnaires will be coded in a standardized manner, and double-entered into our database. Digital measures and recordings will be similarly tracked in our database and regularly backed up. Analyses will be conducted using standard statistical software such as SAS and Matlab.

### Statistical Methods

The primary outcome is PLP indexed by VAS. PLP will be analyzed using intensity of pain over time. To analyze these data, we will initially adopt a mixed analysis of variance (ANOVA) model in which the dependent variable will be the outcome of PLP (such as VAS) and the independent variables will be group (active tDCS-MT; sham tDCS-MT; active tDCS-covered MT; sham tDCS- covered MT), time (baseline and after treatment and follow-up), and the interaction group time. In addition, we will add the random variable ID to account for within participant’s variability and the repeated measures on time. Whenever necessary, post-hoc comparisons with Bonferroni correction for multiple comparisons will be carried out initially to explore significant main effects or interactions. *P* values for secondary and exploratory outcomes will be determined without corrections for multiple comparisons. Furthermore, Pearson’s correlation analyses will be performed to assess the association between PLP relief and changes in non-painful phantom sensations, phantom movements, and telescoping, as measured with VAS. Finally, we will apply a path analysis [60] to the primary outcome data to determine if pain reduction associated with the combined intervention (tDCS plus MT) is due to direct effects versus indirect effects through improvement in secondary outcomes.

We propose that a direct effect of tDCS and MT on PLP can be assumed if the treatment effect cannot be explained by changes in psychological or functional outcomes.

Statistical models for pain will be developed using covariates that include baseline pain, psychological changes, functional changes, and the covariate treatment (main effect of treatment). To complete the path analysis, separate regression models will be run to model the effects of treatment on each outcome alone including all the secondary outcomes. Analyses of the secondary outcomes will be conducted in an exploratory manner (no correction for multiple comparisons). Secondary outcomes are other pain measurements, psychological, neuropsychological and quality of life measurements, and neurophysiological markers (as indexed by TMS and fMRI).

For the intention-to-treat analysis, we will use a conservative method and assume that participants will not improve from the last measured point. We will also perform a sensitive analysis for the missing data using other methods such as completers only.

#### Functional Magnetic Resonance Imaging Data Analysis

Image processing and analysis of functional data will be performed using standard analysis procedures in FSL version 5.0.5 (Oxford Centre for Functional Magnetic Resonance Imaging of the Brain {FMRIB], Oxford University, Oxford, UK). Preprocessing includes head motion correction, B0 unwrapping, brain extraction, intensity normalization, high pass temporal filtering with a frequency cutoff of 120 seconds, and Gaussian spatial smoothing (6.0 mm full width at half maximum). Registration will be performed with FMRIB's Linear Image Registration Tool (FLIRT). Each functional image will be registered to the representative T1-weighted anatomical image (using a 6 degree of freedom boundary based linear registration) and to the MNI 152 template (using a 12-parameter nonlinear affine transformation with a warp resolution of 10 mm). Individual time series analysis will be carried out using a general linear model (GLM). Both active (leg moving and viewing) and passive (rest) conditions will be convolved with a Gaussian hemodynamic response function and their temporal derivatives will be used to model the data. The primary contrasts of interest will be conducted for leg moving versus leg viewing, as well as leg moving versus rest and leg viewing versus rest. The leg moving versus rest condition will be used to identify and define a ROI associated with movement of the contralateral leg during the baseline condition. Activation within this ROI will be compared between pre and post conditions as well as within the same ROI transposed to the opposite hemisphere. As PLP is known to be associated with pathological ipsilateral activation [14,15,19], changes in the degree of lateralization of activation will also be analyzed pre-post in each individual as an index of maladaptive cortical plasticity.

#### Cortical Excitability

TMS data will be analyzed offline using LabChart (ADinstruments, Colorado Springs, CO, USA). Offline analyses will include measures of peak-to-peak amplitude, the area-under-the-curve of all MEPs, ICF, and SICI, and the relative duration of CSPs (time from last MEP until normal muscle activity was re-achieved). We will perform a mixed ANOVA model in which the dependent variable is the measurement of cortical excitability (rMThs, MEPs, SICIs, ICFs, CSPs) and the independent variables are the groups (tDCS active combined with MT active; tDCS active combined with MT sham; tDCS sham combined with MT active and tDCS sham combined with MT sham) and time (pre and post intervention). Furthermore, Pearson’s correlation analyses will be performed to assess the association between PLP relief (VAS change) and cortical excitability changes, as derived from the TMS evaluations.

### Harms

At the end of each stimulation session, participants will complete a side effects questionnaire for tDCS in order to evaluate potential adverse effects of tDCS (tingling, burning sensation, headache, neck pain, mood alterations) and MT (anxiety, grief, dizziness) on a 4-point scale (none, mild, moderate and severe). The participants will be asked whether they have experienced any side effects in an open-ended manner and they will then be specifically asked about headache, neck pain, scalp pain, scalp burns, tingling, skin redness, sleepiness, trouble concentrating, and acute mood change. If any side effects are reported, the degree of relatedness to the intervention will be assessed on a 5-point scale. This type of adverse events questionnaire has been used frequently in our previous tDCS studies [28], including in patients with PLP [61].

In addition, the Side Effects Questionnaire for TMS will be applied at each TMS assessment session. Participants will complete a questionnaire to evaluate potential adverse effects of rTMS (headache, neck pain, itching, and redness at the site of stimulation) on a 5-point scale [62].

### Ethical Considerations

The present study complies with the principles of the Declaration of Helsinki and received approval from the ethics committee (institutional review board, IRB) of Spaulding Rehabilitation Network under the protocol number 2015P001065. Participants will agree to the participation by signing a statement of informed consent. The participants will be allowed to abandon the study at any time with no negative repercussions. All data will be collected in a de-identified manner. Each participant will be identified by an identification number referent to the enroll order. Data will be recorded in hardcopy and electronic form. Hardcopies will be stored in a secured filing cabinet at the administering institution. Electronic copies will be stored in encrypted files on a password-protected computer. All data will be kept for 7 years; following this time, hardcopies will be destroyed by shredding or burning and electronic copies will be deleted by formatting. Participant records will not contain any directly identifiable information.

## Results

This clinical trial received IRB approval in July of 2015 and enrollment started in December of 2015. Currently, 6 participants have been screened and 2 of them successfully met the eligibility criteria. The first participant completed the entire protocol. The second participant is undergoing stimulation sessions. In addition to that, 2 participants are scheduled for screening in the next 2 months. The estimate enrollment rate is about 30 to 35 patients per year; thus we expect to complete enrollment in 4 years.

## Discussion

To the best of our knowledge, this is the first study to combine tDCS and MT for the treatment of PLP. This paper offers a detailed description of a randomized, placebo-controlled, double blind, factorial trial aimed to evaluate the effects of tDCS combined with MT as a rehabilitative tool to decrease PLP, as well as to examine the neural mechanisms underlying PLP in unilateral lower amputees. The results will be published and will provide evidence regarding the use of tDCS combined with MT on this population.

### Study Limitations and Potential Concerns

Some concerns regarding the study design should be discussed. One of them is the choice of using a factorial design; on one hand, this type of design was the best option given that there is no gold standard treatment for PLP. On the other hand, this design increases the number of groups and sample size. For the trial, a conservative approach was used to calculate the sample size in order to avoid a common limitation of clinical trials such as a difficult enrollment and recruitment. It is still possible that our sample size will not be adequate. We do consider that a sample of 132 participants will be appropriate to test our hypothesis, especially taking into account previous effects sizes and the addition of the mechanistic aims (TMS and fMRI).On the other hand, it should be noted that if we find an effect size that is smaller than the one proposed in the study it will not be considered clinically meaningful. This is a 4-year clinical trial; therefore, an expected recruitment rate of 33 subjects per year is appropriate, taking into account the amount of traumatic amputation in the New England area and that the Spaulding Rehabilitation Hospital is a major rehabilitation center for this population. In addition, the factorial design in this trial gives also the possibility of additional secondary analyses between groups. Finally, given the selection of a homogeneous sample (only lower limb and traumatic amputation), it is expected that the study will provide robust results regarding the effects of these treatments in isolation and in combination.

### Anticipated Results

This is a promising clinical trial, given that the previous results reported by our research group showed a decrease in PLP levels indexed by VAS after anodal tDCS when compared with sham [24]. Additional findings showed that multiple sessions of anodal tDCS produced long lasting effects decreasing PLP for up to 2 months [25]. In this context, we anticipate that the combined group therapy (active tDCS and MT) will have a greater decrease on average scores of pain as compared with each therapy alone as well as with the sham/sham group. Furthermore, we expect that the decrease of pain will be correlated with the neurophysiological measurements that will be evaluated with neuroimaging and TMS. With this in mind, we hypothesize that the combined intervention will be able to modulate PLP, and this effect will be correlated with changes of maladaptive plasticity and the amount of cortical reorganization observed after the amputation.

## Abbreviations

ANOVA: analysis of variance
BOLD: blood oxygen level dependent
CoG: center of gravity
CSP: cortical silent period
fMRI: functional magnetic resonance imaging
IRB: institutional review board
MEP: motor-evoked potential
MIR-LEG: movement of the leg observing the mirror
MOV-LEG: movement of the leg
MT: mirror therapy
PLP: phantom limb pain
rMTh: resting motor threshold
ROI: region of interest
SICI: short-interval intracortical inhibition
tDCS: transcranial direct current stimulation
TMS: transcranial magnetic stimulation
VAS: visual analogue scale

## Multimedia Appendix 1

Short presentation with images of intervention.
