# Supplementary material for: Optimizing Rehabilitation for Phantom Limb Pain Using Mirror Therapy and Transcranial Direct Current Stimulation: A Randomized, Double–Blind Clinical Trial Study Protocol
Source: JMIR Res Protoc. 2016 Jul 6;5(3):e138. doi: 10.2196/resprot.5645 (PMC4954918; doi:10.2196/resprot.5645)

Optimizing Rehabilitation for Phantom Limb Pain Using Mirror Therapy and transcranial Direct Current Stimulation (tDCS): A randomized, double – blind clinical trial Study Protocol

Funded by NIH
Clinicaltrials.gov: NCT02487966

SPAULDING
NEUROMODULATION
CENTER

tDCS

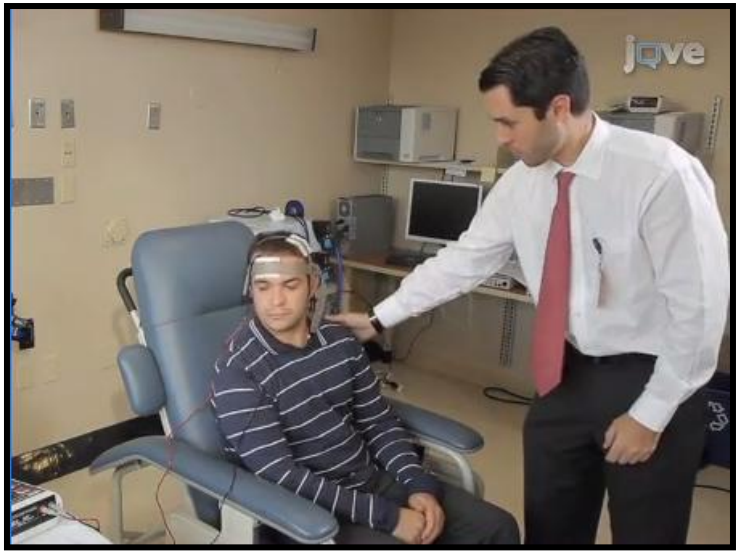

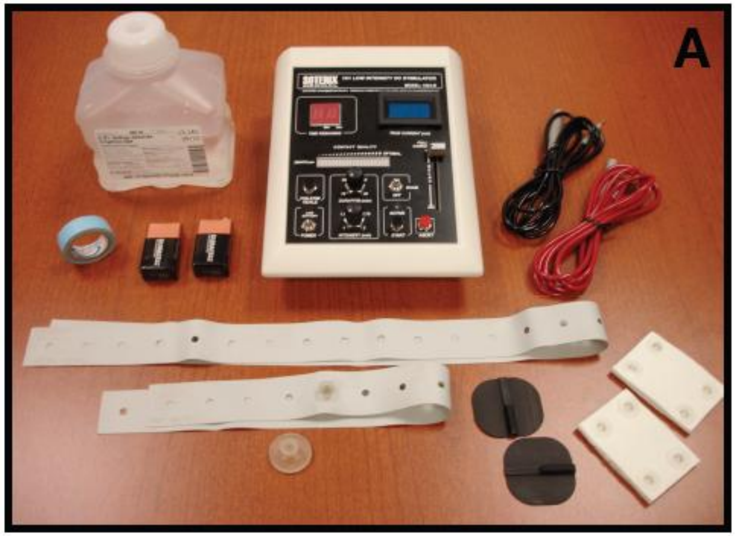

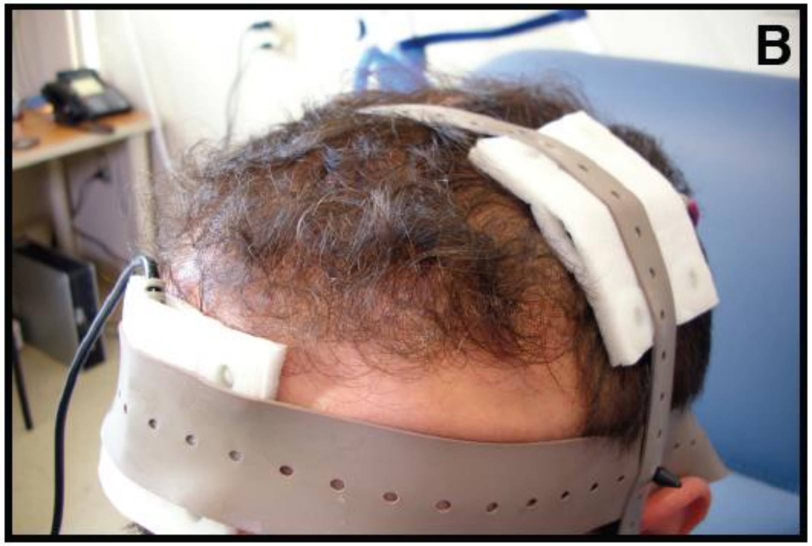

- Primary Aim: To evaluate the efficacy of tDCS and mirror therapy (MT) as rehabilitative tools for the management of pain on chronic PLP patients.

- Secondary Aim: To examine the mechanisms underlying PLP using two neurophysiological techniques.

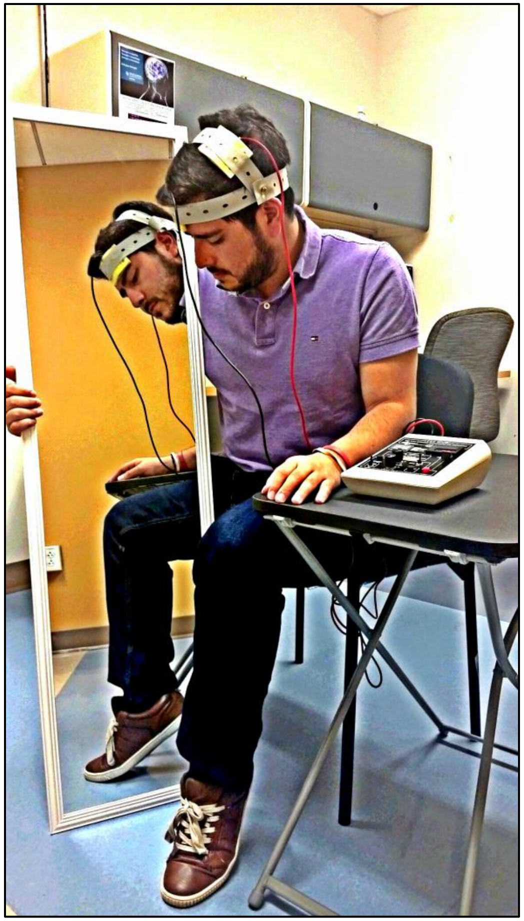

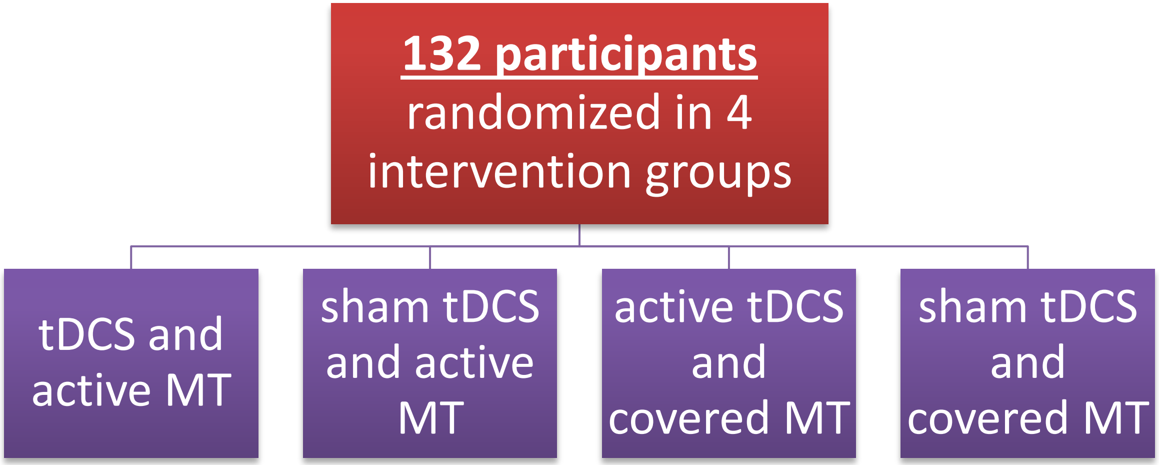

fMRI

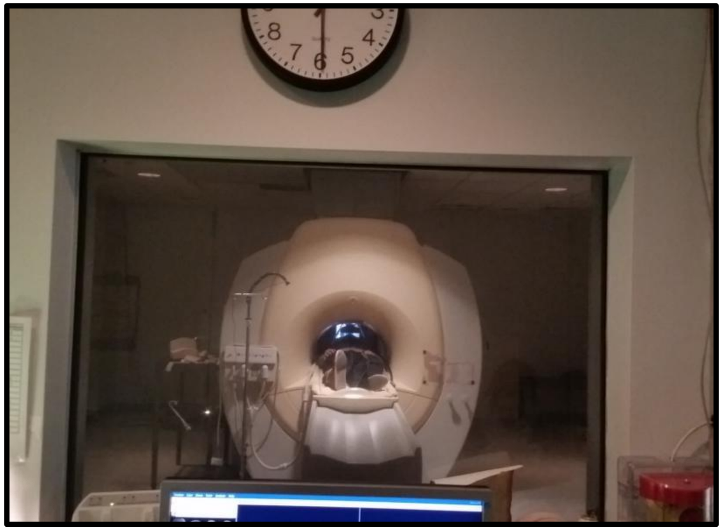

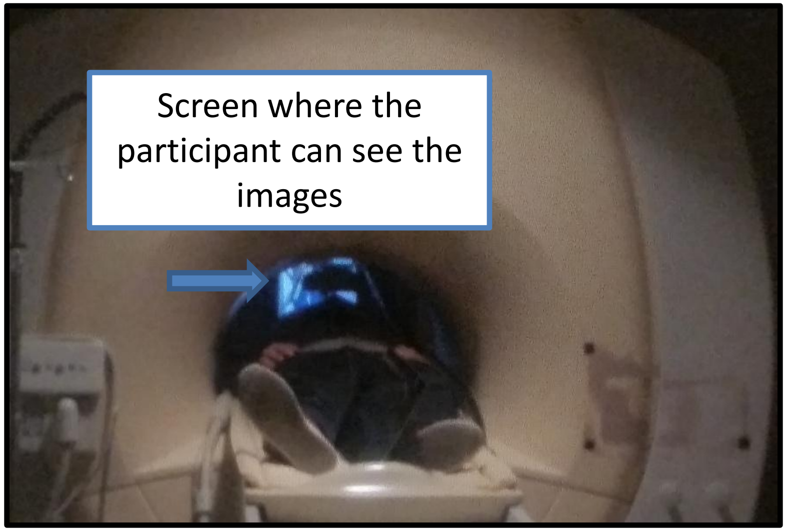
Screen where the participant can see the images

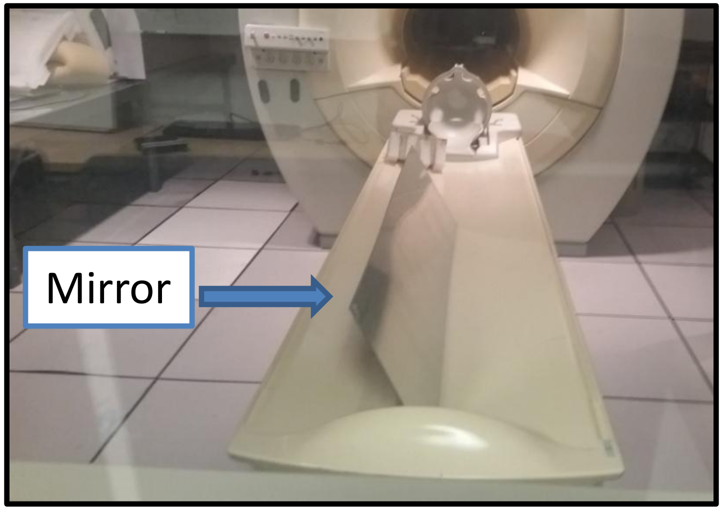
Mirror

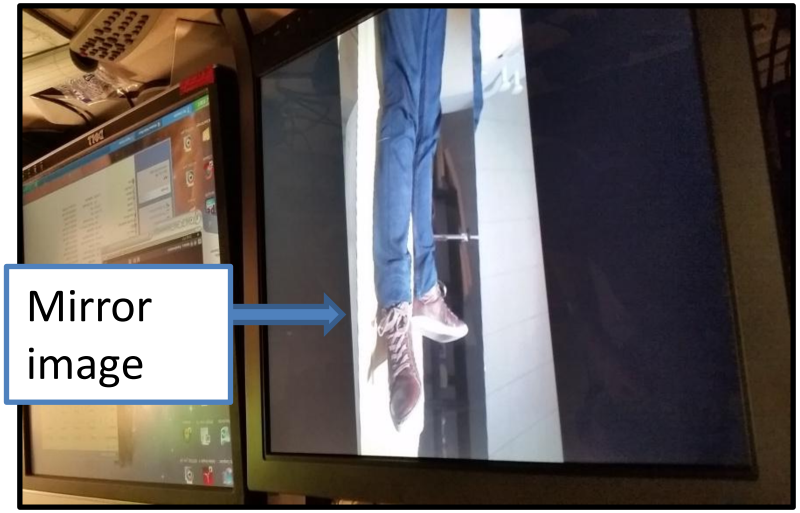
Mirror image

Main Inclusion/Exclusion Criteria

- Older than 18 years
- Unilateral lower limb amputation
- **Traumatic etiology**
- Have chronic phantom limb for at least 3 months
- No metallic implants on head or neck

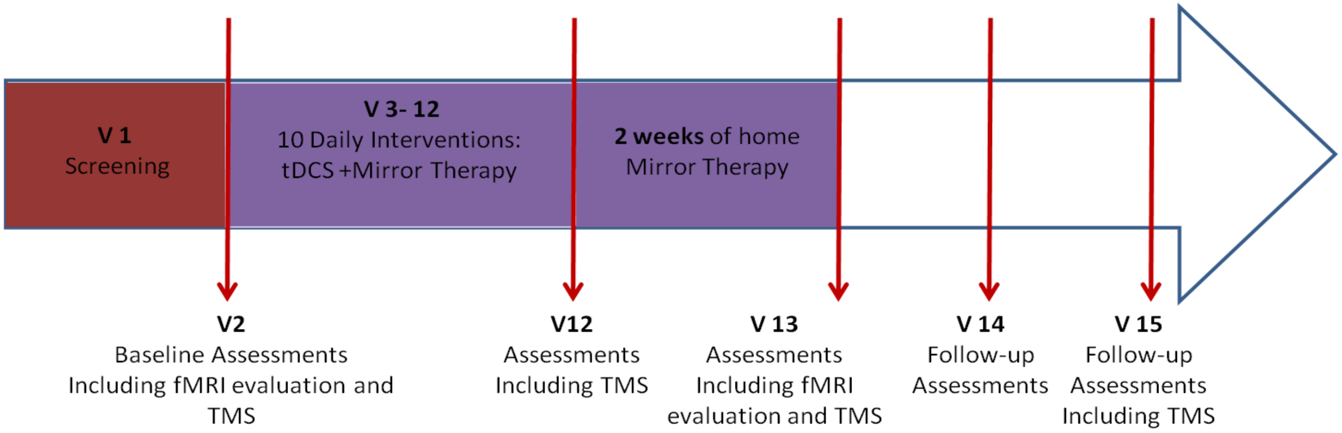

Thank You

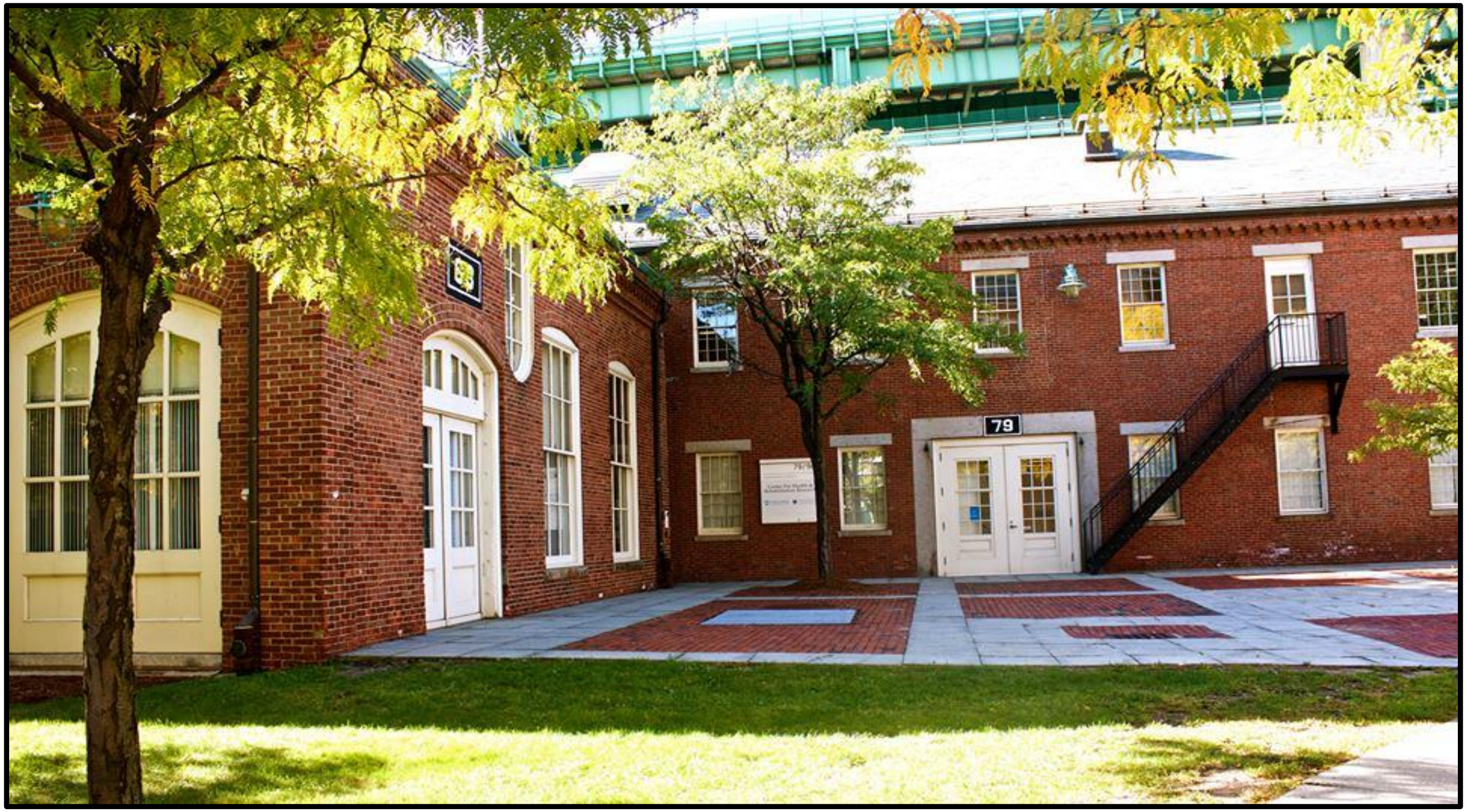

Supplement: Supplementary file 1 [file resprot_v5i3e138_app1.pdf]
